# Chronic disease onset and wellbeing development: longitudinal analysis and the role of healthcare access

**DOI:** 10.1093/eurpub/ckad167

**Published:** 2023-10-06

**Authors:** Barbara Stacherl, Odile Sauzet

**Affiliations:** Socio-economic Panel, German Institute for Economic Research (DIW Berlin), Berlin, Germany; Department of Epidemiology and International Public Health, Bielefeld School of Public Health (BiSPH), Bielefeld University, Bielefeld, Germany; Department of Business Administration and Economics, Bielefeld University, Bielefeld, Germany

## Abstract

**Background:**

Experiencing the onset of a chronic disease is a serious health event impacting living conditions and wellbeing. Investigating wellbeing development and its predictors is crucial to understand how individuals adapt to chronic illnesses. This study (i) analyzed the impact of a chronic disease on wellbeing development, and (ii) explored spatial healthcare access as potential moderating factor.

**Methods:**

Data were obtained from the German Socio-economic Panel, a nationally representative household survey. A prospective sample of 3847 individuals was identified for whom the onset of cancer, cardiopathy, diabetes or stroke was observed between 2008 and 2020. Mixed models using an interrupted time series approach were performed to identify immediate level changes and longitudinal trend changes in wellbeing (operationalized with health and life satisfaction) after disease onset. Further, spatial access to healthcare (operationalized by two-stage floating catchment area measures) as potential moderating factor was examined using interaction effects.

**Results:**

Chronic disease onset had an immediate negative level impact on health and life satisfaction. For health satisfaction, a negative pre-onset wellbeing trend was offset (but not reversed). A small positive trend was observed for life satisfaction after disease onset. Spatial access to healthcare was not associated with the magnitude of wellbeing reduction at onset.

**Conclusions:**

Health and life satisfaction levels drop with the onset of a chronic disease with no recovery trend for health and little recovery for life satisfaction, implying persistently lower wellbeing levels after a chronic illness onset. Spatial access to healthcare does not affect the wellbeing change after disease onset.

## Introduction

Chronic diseases are recognized as major public health challenges due to a steadily rising prevalence (caused by demographic trends and health behaviours) and the burden they pose for individuals.^1^ Chronic diseases are defined as prolonged health issues often accompanied with long-term health limitations and increased care needs, examples being cardiovascular diseases, cancer, diabetes and respiratory diseases.^2^ The onset of a chronic disease marks a serious health event with potential consequences for the wellbeing of patients as they have to adapt to their health condition.^3^^,^^4^

A chronic illness affects many aspects of life. The burden of illness, limitations in daily life, lifestyle changes and/or increased medical needs can pose emotional, psychological, financial and time stressors.^3^^,^^5^ Hence, implications for wellbeing at the time of disease onset as well as changes in the long-term development of wellbeing are expected. The theory of hedonic adaptation states that after a life event, individuals return to a baseline happiness.^6^ Applied to context of chronic illnesses, an immediate reduction in wellbeing at disease onset would be expected, followed by a gradual return to the pre-onset wellbeing levels in the years after the onset. The model of adjusting to chronic conditions, on the other hand, states that after a chronic illness onset, individuals might experience either successful psychological adaptation or ongoing adjustment difficulties.^7^ This implies uncertainty about the long-term development of wellbeing and outcomes ranging from persistently lower wellbeing levels to slow recovery of wellbeing to complete return to pre-onset wellbeing levels might be expected. In the empirical literature, studies across the board found an immediate reduction in subjective wellbeing after a chronic disease onset.^8–12^ Concerning the long-term effects, however, the empirical evidence is more mixed. Some studies reported that subjective wellbeing bounced back after a health shock, implying a return to pre-onset wellbeing in the long term.^11^^,^^13^ Other studies found very little wellbeing recovery, showing persistently lower wellbeing levels after the onset of a chronic disease.^8^^,^^10^ A third stream of studies reported differential outcomes, where some groups achieved more favourable long-term wellbeing development than others.^9^^,^^14^ Given the mixed evidence of previous research, it is of particular interest to investigate the development of wellbeing after illness onset, examining both immediate level changes and longitudinal trend changes. While several studies have analyzed long-term effects of chronic illness by comparing pre- and post-onset wellbeing levels,^8^^,^^10^^,^^12^ longitudinal analyses including both immediate level and long-term trend effects are scarce.^14^ However, joint analyses of level and trend effects are crucial to disentangle immediate and long-term impacts of a chronic disease onset.

With the onset of a chronic disease often follow increased medical needs and higher health service utilization requiring a patient’s effort to manage their disease. This work of managing a chronic disease, including, e.g. administering medication or arranging and attending medical appointments, is referred to as the burden of treatment.^5^ Low availability of healthcare services or long distances to healthcare providers can be perceived as burdensome for persons with chronic diseases.^15^ Against this background, lower accessibility and availability of healthcare services might aggravate this treatment burden. Put differently, worse spatial healthcare access might amplify wellbeing reduction at disease onset. To the best of our knowledge, no studies to date have investigated spatial healthcare access as potential moderating effect in the relationship of chronic illness onset and subjective wellbeing.

This study investigates the development of individual wellbeing around the onset of a chronic disease and contributes to the literature in two main ways. The first goal of this study is to disentangle immediate wellbeing level changes and longitudinal wellbeing trend changes following an observed onset of a chronic disease. To that end, within-person changes in levels and trends of health and life satisfaction due to the onset of cancer, cardiopathy, diabetes or stroke are investigated. The second goal of this study is to examine the role of accessibility and availability of healthcare services for wellbeing after chronic illness onset. Spatial access to healthcare, operationalized by a two-step floating catchment area method, is investigated as a potential moderating factor for the impact of chronic illness onset on wellbeing.

## Methods

### Data sources

This study drew population data from the German Socio-Economic Panel (SOEP), a representative longitudinal survey of private households in Germany. The SOEP was started in 1984 and has been conducted yearly. It includes around 30 000 individuals in over 15 000 households. The core survey population covers individuals from age 17 onward representing the resident population in all German federal states.^16^ Information on chronic illnesses is surveyed bi-annually since 2009. Questions on overall and domain-specific life satisfaction appear in all waves. To add information on healthcare access, health services location data from hospitals (data: Krankenhausverzeichnis, provider: Statistisches Bundesamt) and outpatient physicians (data: Bundesarztregister, provider: Kassenärztliche Bundesvereinigung) were linked to the survey data.

### Data description

#### Sample

The study sample consisted of individuals for whom the onset of a chronic illness was observed. Self-reports on diagnosed diseases were captured in the SOEP via the question: ‘Has a doctor ever diagnosed you to have one or more of the following illnesses’ with a range of diseases as possible responses. Our sample thus consisted of individuals who reported never having been diagnosed with a certain disease when first asked and indicated having been diagnosed with that same disease in a later wave. If more than one illness onset was observed sequentially, only the first incidence was used. Based on a definition of frequent chronic diseases,^2^ four illnesses were chosen for investigation: cancer, cardiopathy, diabetes and stroke. This resulted in a sample of 3847 individuals for whom a chronic disease onset and wellbeing development (= at least two observations before and after onset) could be observed in 2008–20. Sample statistics are presented in table 1.

**Table 1:** Sample characteristics

	Total	Cancer	Cardiopathy	Diabetes	Stroke	Multiple
Individuals, *N*						
	3847	867	1531	851	352	246
Observations per individual, mean (SD)^a^						
Before	4.5 (2.4)	4.6 (2.4)	4.5 (2.4)	4.4 (2.3)	4.6 (2.5)	4.3 (2.3)
After	5.0 (2.5)	5.0 (2.6)	5.0 (2.5)	5.2 (2.6)	4.8 (2.4)	4.9 (2.4)
Health satisfaction, mean (SD)^a^						
Before	6.0 (1.9)	6.3 (1.9)	6.0 (1.9)	5.9 (1.9)	5.5 (2.2)	5.5 (2.0)
After	5.3 (2.0)	5.4 (2.1)	5.4 (1.9)	5.5 (1.9)	4.7 (2.1)	4.8 (1.8)
Life satisfaction, mean (SD)^a^						
Before	7.0 (1.5)	7.2 (1.4)	7.0 (1.5)	6.9 (1.5)	6.7 (1.7)	6.7 (1.7)
After	6.8 (1.6)	7.0 (1.6)	6.9 (1.6)	6.8 (1.6)	6.5 (1.8)	6.4 (1.8)
Age at onset, mean (SD)^b^						
	62.2 (14.3)	61.5 (13.6)	63.0 (14.7)	58.7 (13.8)	67.1 (14.4)	64.0 (13.2)
Gender, *N* (%)						
Male	1869 (48.6%)	370 (42.7%)	761 (49.7%)	412 (48.4%)	198 (56.2%)	128 (52%)
Female	1978 (51.4%)	497 (57.3%)	770 (50.3%)	439 (51.6%)	154 (43.8%)	118 (48%)
Health insurance, *N* (%)^b^						
Public	3350 (87.1%)	723 (83.4%)	1320 (86.2%)	771 (90.6%)	309 (87.8%)	227 (92.3%)
Private	497 (12.9%)	144 (16.6%)	211 (13.8%)	80 (9.4%)	43 (12.2%)	19 (7.7%)
Doctor visits in last 3 months, mean (SD)^b^						
	4.5 (6.1)	5.3 (6.7)	4.1 (5.1)	3.6 (4.1)	5.0 (8.0)	6.3 (10.3)
Hospital stays in last year, mean (SD)^b^						
	0.6 (1.4)	0.8 (1.5)	0.5 (1.2)	0.3 (0.7)	0.7 (0.9)	1.1 (3.5)
2SFCA hospitals, mean (SD)^b^						
	6.4 (8.8)	6.3 (8.0)	6.4 (8.9)	6.4 (9.6)	6.2 (7.2)	6.7 (9.7)
2SFCA general practitioners, mean (SD)^b^						
	6.6 (5.1)	6.7 (5.4)	6.5 (5.1)	6.7 (5.5)	6.3 (4.3)	6.3 (4.5)

a Averaged within individual, then within illness group to avoid pooling bias.

b Measured in first year after onset.

#### Outcome

The outcome of interest, wellbeing, was operationalized with two satisfaction measures: domain-specific satisfaction with health, and current overall life satisfaction. Life and health satisfaction were measured with one item each and surveyed every year. Study participants were asked ‘How satisfied are you with your health?’ regarding health satisfaction, and ‘How satisfied are you with your life, all things considered?’ regarding life satisfaction. For both items, ranking on an 11-point scale was required, reaching from 0 (completely dissatisfied) to 10 (completely satisfied).

#### Onset effects

Of interest for the first research goal was the effect of chronic illness onset on life and health satisfaction, both in immediate (level change) and longitudinal (trend change) terms. To investigate the immediate and longitudinal effects, we used a dummy variable indicting pre- and post-onset years and a relative time (in years) variable centred around the onset, respectively. Chronic illnesses were captured in the SOEP in terms of lifetime prevalence. This allowed for indirect identification of the time of disease onset through an observed switch in lifetime prevalence (from 2009 to 2011; from 2011 to 2013; etc.). Periods in which respondents reported never having been diagnosed with a certain disease were categorized as pre-onset. All periods from the first self-report of being diagnosed with that same disease were categorized as post-onset. As illness diagnoses were surveyed bi-annually, diagnosis status could not be determined for one wave in between last pre-onset and first post-onset period, thus this observation was dropped.

#### Access effect

Considering the second goal, spatial access to healthcare services was the exposure of interest. Spatial healthcare access was operationalized using a two-stage floating catchment area (2SFCA) method, a well-established measure to quantify spatial healthcare access which integrates both accessibility (i.e. distance) and availability (i.e. capacity).^17^^,^^18^ It is computed in two steps: (i) for each provider point, all population points within a catchment area are summed up and weighted by distance, creating a provider-to-population ratio; and (ii) for each population point, all such provider-to-population ratios are summed up (again, weighted by distance). Thus, the 2SFCA reflects individualized spatial access (rather than a regional aggregate) and accounts for distance decay i.e. that far away providers are less accessible. Metrics were computed for access to hospitals and to general practitioners (GPs). Similar to a physician density, the hospital 2SFCA measure is interpreted in terms of hospital beds per 1000 inhabitants and the GP 2SFCA is interpreted in terms of GP full-time equivalents per 10 000 inhabitants. A detailed description of how the 2SFCA measures were computed is given in the Supplementary material.

#### Covariates

To control for potential confounding factors, demographic, socioeconomic and health-related covariates were included. Specifically, these were: age at onset, age at onset squared, employment situation, gender, household type, income, number of doctor visits, number of hospital stays, number of years in study sample, relocation and type of health insurance. Continuous variables were mean standardized.

### Statistical analyses

#### Onset effects

To evaluate the first goal, we investigated the impact of disease onset on immediate level changes and longitudinal trend changes for life satisfaction and health satisfaction using a mixed model with an interrupted time series approach (ITSA). ITSA is commonly used when no suitable control group can be identified and allows to disentangle a ‘level’ effect (immediate level change at onset) and a ‘trend change’ effect (change in trend after onset).^19^ This was used to capture within-person changes following the illness onset. To allow for the investigation of within-person changes in the wellbeing trend (rather than sample averages), regression analyses were conducted with the restriction that after exclusion of missing values, at least two pre-onset and two post-onset observations be present per individual. Individual level random intercepts and random slopes were used to account for the data structure entailing multiple measures per individual and to avoid sample composition bias. The model took the following form:

$$y_{it}=\;\beta_{0}+\;\beta_{1}.T_{it}+\;\beta_{2}.O_{it}+\;\beta_{3}.T_{it}.O_{it}+\;\beta_{k}.X_{kit}+u_{oi}+u_{1i}.T_{it}+u_{2i}.O_{it}+u_{3i}.T_{it}.O_{it}+\varepsilon_{it},$$

where $T_{it}$ is the relative time-to-onset variable, $O_{it}$ is a binary variable indicating pre- and post-onset periods for individual i in year t, $T_{it}.O_{it}$ is the interaction of time-to-onset*onset, $X_{kit}$ is the set of k control variables as outlined above, $u_{0i}$ is the random intercept for individual i and $u_{1i}$, $u_{2i}$ and $u_{3i}$ are random slopes for individual i. Hence, $\beta_{1}$ describes the pre-onset time trend, $\beta_{2}$ describes the immediate level effect and $\beta_{3}$ captures the post-onset trend change. Analyses were conducted for life and health satisfaction.

#### Access effect

To evaluate the second goal, a potential moderating effect of spatial healthcare access for the impact of illness onset on wellbeing was examined. We modelled an interaction of the onset indicator with a spatial healthcare access indicator to explore if access affects the magnitude of the wellbeing reduction at disease onset. Two terms were introduced into the above outlined regression model—one for spatial access to hospitals (2SFCA hospitals), and one for spatial access to GPs (2SFCA GPs), each interacted with the onset dummy. This was conducted for both health satisfaction and life satisfaction.

All analyses were conducted in R, version 4.2.2.^20^ The ‘tidyverse’ package^21^ was used for data manipulation, the ‘sf’ package^22^ was used for computation of spatial access measures and the ‘lme4’ package,^23^ in particular the ‘lmer’-function, was used to fit the mixed models.

#### Sensitivity analyses

For robustness, we (i) conducted the regression analyses with random intercepts and explanatory variables at the district level to account for potentially unobserved regional effects and (ii) used alternative operationalizations for potential spatial healthcare access (distances to nearest hospital and GP, hospital bed/GP density within 10/3 km).

## Results

### Sample characteristics

**Figure 1:**
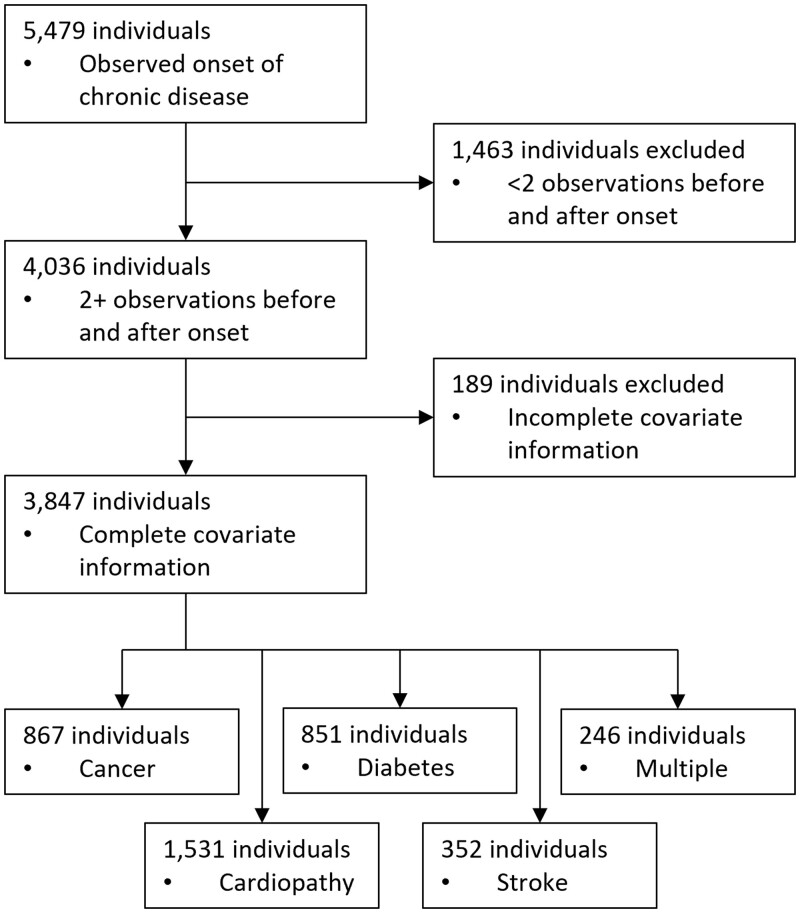
Flow chart of sample identification process

We identified 5479 individuals for whom the onset of either cancer, cardiopathy, diabetes or stroke could be observed in the data. At least two observations before and two observations after disease onset in the time period 2008–20 were needed to estimate individual-specific time trends and trend changes—4036 individuals fit these criteria. Due to incomplete covariate information leading to an insufficient number of observations per individual, another 189 individuals were dropped leaving us with a final unbalanced sample including 36 463 observations of 3847 individuals. This corresponded to 867 cancer, 1531 cardiopathy, 851 diabetes, 352 stroke and 246 multiple (in same year) onsets occurring between 2008 and 2020. The sample identification process is depicted in figure 1. On average, 4.5 (SD 2.4) observations per person were recorded before illness onset and 5.0 (SD 2.5) observations after onset. The mean age at onset was 62.2 (SD 14.3), 51.4% (*N* = 1978) of the sample were female. Regression results are reported in table 2.

**Table 2:** Regression results—chronic illness onset and wellbeing

	Health satisfaction						Life satisfaction					
	Onset model			Access model			Onset model			Accsess model		
	Estimate	95% CI	*P*-value	Estimate	95% CI	*P*-value	Estimate	95% CI	*P*-value	Estimate	95% CI	*P*-value
Intercept	6.060	5.904 to 6.217	<0.001	6.061	5.904 to 6.217	<0.001	6.478	6.346 to 6.611	<0.001	6.478	6.345 to 6.610	<0.001
Time-to-onset	−0.059	−0.073 to −0.045	<0.001	−0.059	−0.073 to −0.045	<0.001	0.004	−0.007 to 0.015	0.469	0.004	−0.007 to 0.015	0.471
Onset: yes (no)	−0.383	−0.452 to −0.314	<0.001	−0.383	−0.452 to −0.314	<0.001	−0.184	−0.239 to −0.129	<0.001	−0.184	−0.239 to −0.129	<0.001
Time-to-onset*	0.047	0.028 to 0.065	<0.001	0.047	0.028 to 0.066	<0.001	0.026	0.011 to 0.041	<0.001	0.026	0.011 to 0.041	<0.001
Onset: yes (no)												
2SFCA hospitals* Onset: yes (no)				0.010	−0.037 to 0.057	0.670				0.010	−0.028 to 0.048	0.596
2SFCA GPs*				−0.046	−0.093 to 0.001	0.057				0.006	−0.032 to 0.043	0.770
Onset: yes (no)												
Control variables	Yes			Yes			Yes			Yes		
Random intercept	Yes			Yes			Yes			Yes		
Random slopes	Yes			Yes			Yes			Yes		
*N* observations	36 463			36 463			36 463			36 463		
*N* individuals	3847			3847			3847			3847		

Notes: Time-to-onset = pre-onset trend effect; Onset: yes = immediate level effect at onset; Time-to-onset*Onset: yes = post-onset trend change effect. Continuous variables are mean standardized. 95% CI, 95% confidence interval; 2SFCA, two-step floating catchment area method; GP, general practitioner.

**Figure 2:**
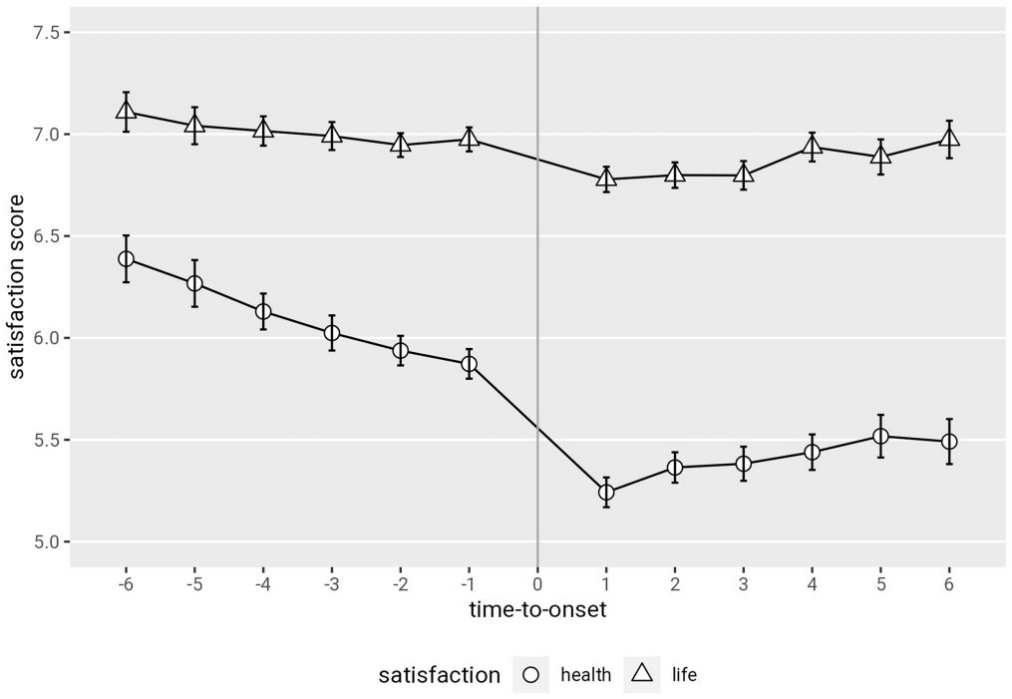
Life and health satisfaction development with time-to-onset. Note: Observation in *t*_0_ omitted, because for this time point it is unknown whether chronic illness is present or not (data on self-reported illness diagnoses collected bi-annually). Displayed time range represents maximum before and after onset observations possible in study period (2008–20)

Visual inspection of unadjusted time-to-onset developments indicate a dip in both life and health satisfaction at disease onset (see figure 2), with the dip in health satisfaction being more pronounced. Further a slight positive trend after illness onset is seen, albeit at a lower level than before onset.

### Onset effects

The pre-onset trend in health satisfaction reflected by the time-to-onset variable was negative (pre-onset trend estimate: −0.059, 95% CI: −0.073 to −0.045) indicating lower satisfaction with health already before disease onset. The onset of a chronic disease was associated with an immediate level effect—a significant drop in health satisfaction (onset level estimate: −0.383, 95% CI: −0.452 to −0.314) at the time of illness onset was observed. With illness onset, a positive change in the wellbeing trend was observed (post-onset trend change estimate: 0.047, 95% CI: 0.028 to 0.065), reflected by the ‘time-to-onset * onset’ interaction term. The effect size of the post-onset trend change indicates that the negative pre-onset trend was largely offset, however, the ‘trend change’ did not result in in an overall positive trend, implying a durable health satisfaction reduction. Looking at overall life satisfaction, illness onset was also associated with a significant reduction in satisfaction (onset level estimate: −0.184, 95% CI: −0.239 to −0.129), however, the immediate level change is smaller for life than for health satisfaction. Pre-onset development of life satisfaction did not show a significant trend effect (pre-onset trend estimate: 0.004, 95% CI: −0.007 to 0.015). The post-onset trend change in life satisfaction was positive but small (post-onset trend change estimate: 0.026, 95% CI: 0.011 to 0.041). With a zero trend before onset and a small positive ‘trend change’ after onset this implies a small positive trend for life satisfaction after disease onset. Regression results were stable in terms of direction and significance of effects across the above outlined robustness specifications.

### Access effect

The potential moderating effect of spatial access to healthcare is reflected by the ‘2SFCA hospitals * onset’ and the ‘2SFCA GPs * onset’ interaction terms. The magnitude of the immediate level effect of disease onset on health satisfaction was neither associated with spatial access to hospitals (estimate: 0.010, 95% CI: −0.037 to 0.057), nor with that to GPs (estimate: −0.046, 95% CI: −0.093 to 0.001), implying that lower spatial access to hospitals and GPs did not amplify the reduction in health satisfaction at onset. Equally for life satisfaction, neither spatial access to hospitals (estimate: −0.010, 95% CI: −0.028 to 0.048) nor to GPs (estimate: 0.006, 95% CI: −0.032 to 0.043) was associated with the level reduction at disease onset. The null hypothesis of healthcare access not being related to the wellbeing level change at disease onset thus cannot be rejected.

## Discussion

In this longitudinal prospective study, we investigated the impact of a chronic disease onset on wellbeing for over 3800 individuals in Germany. We examined immediate and longitudinal changes in health and life satisfaction following the onset of cancer, cardiopathy, diabetes or stroke. An immediate drop in both health and life satisfaction was seen. For the long-term, our results imply durable reductions in wellbeing following a chronic illness onset with only limited recovery trends. Spatial healthcare access to hospitals and GPs was not related to wellbeing reduction at disease onset, i.e. lower spatial healthcare access did not seem to increase patients’ burden of treatment.

The finding that individual wellbeing was reduced immediately after the onset of a chronic illness is in line with previous literature.^10–12^^,14^ In this study, the wellbeing drop was observed both for health and life satisfaction and held when controlling for demographic, socioeconomic and health-related variables. The immediate level effect was larger for health satisfaction than for life satisfaction. These findings confirm a wellbeing reduction associated with disease onset, across multiple chronic diseases.

Complementing earlier research, we further investigated wellbeing development over time. We found a negative wellbeing trend prior to disease onset for health satisfaction but not for life satisfaction. This pre-onset time trend might point to symptoms experienced already prior to disease diagnosis,^24^ or to age effects. After disease onset, we found a positive trend change for health satisfaction, which largely offset the previously negative trend but did not lead to a positive overall post-onset trend. Taken together, this suggests a negative trend in health satisfaction before disease onset, a drop in satisfaction at onset and persistently (during the study period) lower health satisfaction levels after onset. We found no pre-onset trend effect and a small positive post-onset trend effect for life satisfaction. That is, contrary to health satisfaction, life satisfaction was not reduced before disease onset, and followed a recovery path after disease onset. Considering the magnitude of the life satisfaction drop at disease onset, however, the results suggest only limited recovery of the life satisfaction lost at disease onset. Thus, for health nor life satisfaction persistent wellbeing reductions triggered by a chronic disease onset were observed. These findings are similar to a previous study on disability onset, which showed very little adaptation in terms of life satisfaction over time.^25^ Our results therefore suggest no substantial wellbeing recovery when looking at the longer-term effects of chronic disease. This could point either to persistent physical limitations (i.e. limited recovery of health status) affecting wellbeing or to persistent emotional and psychological burdens connected with the chronic disease (i.e. limited psychological adaptation). These findings run counter to the hedonic adaptation theory,^6^ which states that individuals return to a baseline happiness after bad life events, and fit better with Moss-Morris’ conceptualization of chronic illness adaptation,^7^ which entails adjustment difficulties as possible outcome. It is noted here that our data included a maximum of nine post-onset years, and we cannot rule out that wellbeing recovery occurs at later time points.

To increase our understanding of the role of healthcare access for individuals with a chronic illness, we examined whether spatial access to healthcare services had an effect on the magnitude of the immediate wellbeing reduction at disease onset. This was to test whether lower availability of healthcare and longer distances to providers would aggravate the treatment burden perceived. Contrary to our hypothesis, we found no aggravating effect of lower spatial access to healthcare for the wellbeing drop. This was true for access to hospitals and to GPs and both for health and life satisfaction. Thus, our findings do not suggest that individuals with worse spatial healthcare access experience a higher treatment burden resulting in larger wellbeing reductions. Further analyses are needed to build up comprehensive knowledge on factors mediating wellbeing reductions at chronic disease onset.

There are some limitations to our analyses. Firstly, as any study analyzing health outcomes longitudinally, this study might be subject to survival bias. As individuals who did not participate due to health restrictions or death naturally were not represented in our sample, we cannot make any inference on this group. Secondly, individuals’ wellbeing developments might be heterogeneous across illness groups. We conducted joint analyses for individuals with cancer, cardiopathy, diabetes or stroke to benefit from a large sample size and to unravel common patterns that describe wellbeing development for this broad group. Future studies should aim to investigate heterogeneities across illnesses.

A major strength of this study is its use of prospective longitudinal data, allowing for identification of disease onsets and longitudinal analysis of pre- and post-onset wellbeing development. We used a large, population-based sample of 3847 individuals for whom a chronic disease onset was observed in the period 2008–20. The questionnaire data were combined with healthcare services location data to account for individual access to healthcare. Further, this study was able to disentangle immediate and longitudinal wellbeing effects provoked by a chronic disease onset. Thus, level and trend changes could be analyzed separately.

From a policy perspective, additional efforts are required to target wellbeing around the onset of a chronic disease. Ensuring that people with a chronic disease receive appropriate support to navigate the adaptation process to their new health condition is required. Regarding healthcare access, we find no evidence that lower spatial access aggravates the wellbeing level drop at disease onset. While accessing healthcare services itself may be perceived as burdensome, comparatively worse spatial access does not seem to translate into lower overall wellbeing around the chronic disease onset, at least not in the German context where healthcare access is generally good. Future research should investigate the role of spatial access in the long run as individuals with a chronic disease continue to require healthcare services possibly throughout their life. Our study provides evidence that chronic disease onset plays a substantial role for wellbeing and highlights the importance of being able to differentiate between immediate level and longitudinal trend effects.

## Supplementary Material

ckad167_Supplementary_Data

## Data Availability

The data underlying this article will be shared on reasonable request to the corresponding author. Key points This study investigates longitudinal wellbeing effects of chronic illness. Health and life satisfaction levels drop with the onset of a chronic disease. We find no clear recovery trend for health satisfaction and limited recovery over time for life satisfaction. Chronic disease onset triggers persistent wellbeing reductions. Spatial access to healthcare does not moderate the wellbeing reduction at disease onset.
