# Supplementary material for: Chronic disease onset and wellbeing development: longitudinal analysis and the role of healthcare access
Source: Eur J Public Health. 2023 Oct 6;34(1):29–34. doi: 10.1093/eurpub/ckad167 (PMC10843952; doi:10.1093/eurpub/ckad167)
Supplement: ckad167_Supplementary_Data [file ckad167_supplementary_data.docx]

To operationalize an individual's spatial access to healthcare we used a two-stage floating catchment area (2SFCA) method. 2SFCA measures are widely used in health research to quantify spatial healthcare access. The 2SFCA intuition is similar to a physician density in that it captures provider-to-population ratios, however, it has two main advantages: First, it is not a regional aggregate but depicts spatial access at a specific location and thus also accounts for potential border crossing in healthcare use. Second, it integrates availability and accessibility by incorporating both the provider-to-population ratio and distance decay (decreasing access with increasing distance) effects. The 2SFCA method was first presented by Luo & Wang (2003) and consists of two steps:

1. Around each provider location $j$, all population locations $k$ within a predefined catchment area (often defined as 30 minutes driving time) are searched. The provider capacity (e.g., number of physicians) is divided by the total population within the catchment area to create a location-specific provider-to-population ratio. This takes on the form:

$$R_{j}= \frac{S_{j}}{\sum_{k \in\{d_{kj}\leq d_{o}\}} P_{k}}$$

where$R_{j}$ is the provider-to-population ratio at location $j$, $S_{j}$ is the provider capacity at location $j$, $P_{k}$ is the population at location $k$, $d$ is the distance between healthcare provider and population location, and $d_{0}$ is the distance threshold defining the catchment area.

1. Around each population location $i$, all provider locations $j$ within the same predefined catchment area are searched. The provider-to-population ratios of all provider locations within the catchment area are summed up to build the spatial access measure. This takes on the form:

$$A_{i}= \sum_{j \in\{d_{ij}\leq d_{o}\}} R_{j} =\sum_{j \in\{d_{ij}\leq d_{o}\}} \frac{S_{j}}{\sum_{k \in\{d_{kj}\leq d_{o}\}} P_{k}}$$

Since its initial introduction, many methodological advancements have been proposed to the 2SFCA method. Most notably, continuous (instead of binary) distance decay functions have been introduced to reflect varying accessibility also within the catchment area. Wang (2012) proposed a generalized 2SFCA method which adds a generic distance decay function $f(d)$ in both steps.

This study built on the generalized 2SFCA method, applying a negative power function, more specifically, an inverse distance decay function. This took on the form:

$$A_{ip}= \sum_{j \in\{d_{ij}\leq d_{o}\}} \frac{S_{j} d_{ij}^{-\beta}}{\sum_{k \in\{d_{kj}\leq d_{o}\}} P_{k} d_{kj}^{-\beta}}$$

where $A_{ip}$ is the spatial accessibility at location $i$ for provider type $p$, $S_{j}$ is the capacity (for hospitals: hospital beds, for GPs: GP full-time equivalents) of the healthcare provider at location $j$, $P_{k}$ is the population at location $k$, $d$ is the distance between healthcare provider and population location, and $d^{-\beta}$ reflects the negative power distance decay function. In our study the friction coefficient $\beta$ took on a value of 1, thus the distance decay function corresponded to an inverse distance weighting. We assumed equal travel friction for all distances below 100m to avoid extreme inverse distance weights for very close proximity (e.g., doctor’s practice at respondent address).

This measure was applied once for access to hospitals with $d_{0}$ = 10km and once for access to general practitioners with $d_{0}$ = 3km. The catchment area definition was based on the Euclidean (aerial) distance. We relied on small-area level census data (100mx100m grid) to depict the population distribution. For ease of interpretation, we defined the provider-to-population ratio per 1,000 inhabitants for hospitals and per 10,000 inhabitants for general practitioners. We used census data from the year 2011 as this was the only year in our study period for which the fine-grained population data were available. Current provider data on hospital locations and GP locations (geocoded street addresses) were used for 2009-2019. Thus, we computed for each individual in our study sample the spatial access to hospitals as well as the spatial access to general practitioners for each year. The 2SFCA for hospitals is to be interpreted as follows: Number of hospital beds per 1,000 inhabitants within a 10km radius around the individual, demand-adjusted and distance weighted. The 2SFCA for general practitioners is to be interpreted as follows: Number of general practitioner full-time equivalents per 10,000 inhabitants within a 3km radius around the individual, demand-adjusted and distance weighted.

Figure S1: Health satisfaction development with time-to-onset by illness

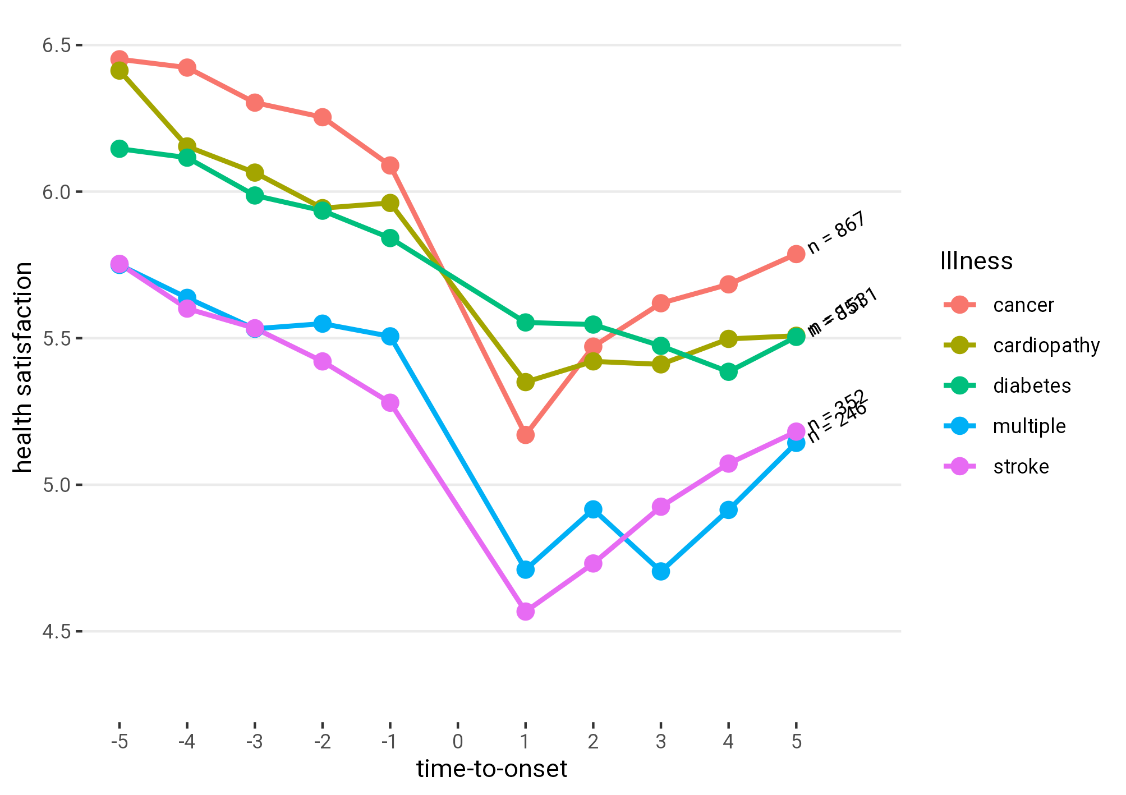

*Note*: Observation in t_0_ omitted, because for this time point it is unknown whether chronic illness is present or not (data on self-reported illness diagnoses collected bi-annually). Displayed time range capped for legibility.

Figure S2: Life satisfaction development with time-to-onset by illness

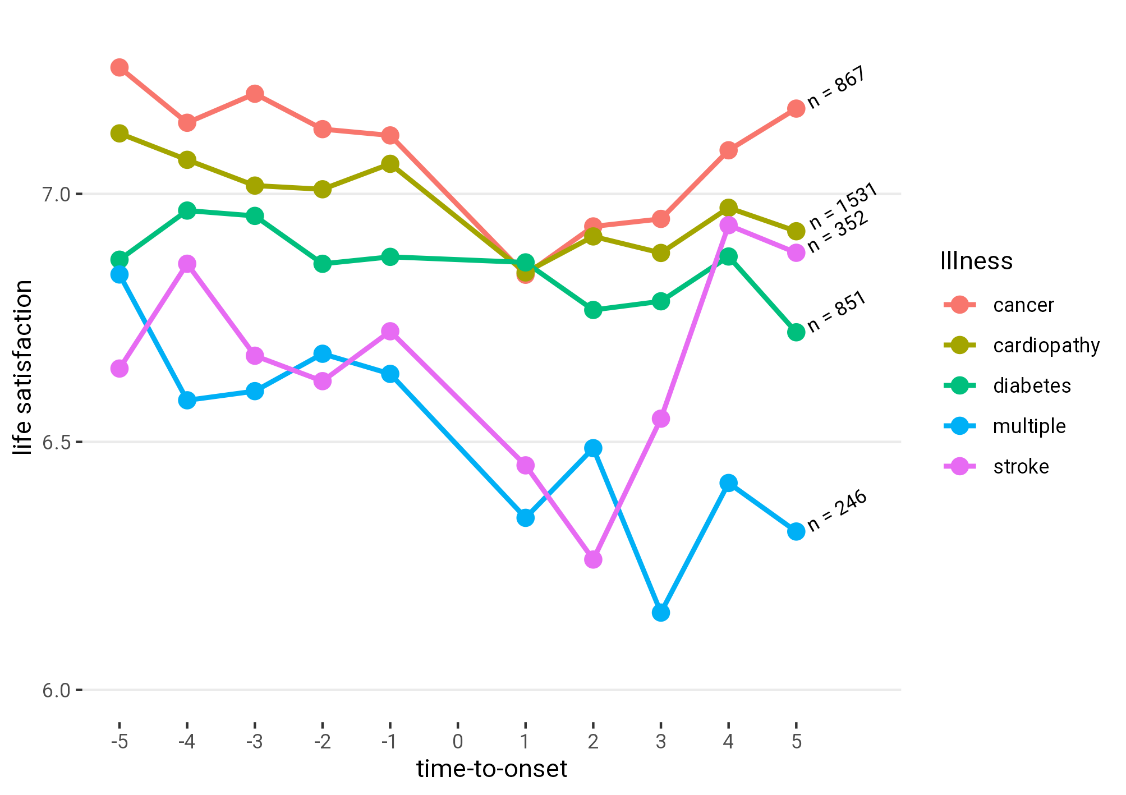

*Note*: Observation in t_0_ omitted, because for this time point it is unknown whether chronic illness is present or not (data on self-reported illness diagnoses collected bi-annually). Displayed time range capped for legibility.

Table S1: Full Regression Results – Health Satisfaction

|  | **Onset Model** | | | **Access Model** | | |
| --- | --- | --- | --- | --- | --- | --- |
|  | **Estimate** | **95%-CI** | **p-value** | **Estimate** | **95%-CI** | **p-value** |
| Intercept | 6.060 | 5.904– 6.217 | <0.001 | 6.061 | 5.904– 6.217 | <0.001 |
| **Time-to-onset** | -0.059 | -0.073–-0.045 | <0.001 | -0.059 | -0.073–-0.045 | <0.001 |
| **Onset: yes [no]** | -0.383 | -0.452–-0.314 | <0.001 | -0.383 | -0.452–-0.314 | <0.001 |
| **Time-to-onset*Onset: yes [no]** | 0.047 | 0.028– 0.065 | <0.001 | 0.047 | 0.028– 0.066 | <0.001 |
| Age | -1.308 | -1.653–-0.962 | <0.001 | -1.306 | -1.652–-0.961 | <0.001 |
| Age² | 1.070 | 0.723– 1.416 | <0.001 | 1.069 | 0.723– 1.415 | <0.001 |
| Sex: female [male] | -0.097 | -0.199– 0.004 | 0.061 | -0.098 | -0.200– 0.004 | 0.060 |
| Years in sample | 0.139 | 0.092– 0.185 | <0.001 | 0.139 | 0.092– 0.185 | <0.001 |
| Year of onset: 2013 [2011] | 0.000 | -0.159– 0.160 | 1.000 | 0.000 | -0.159– 0.160 | 1.000 |
| Year of onset: 2015 [2011] | 0.117 | -0.027– 0.261 | 0.112 | 0.118 | -0.027– 0.262 | 0.110 |
| Year of onset: 2017 [2011] | 0.192 | 0.035– 0.350 | 0.017 | 0.192 | 0.035– 0.350 | 0.017 |
| Year of onset: 2019 [2011] | 0.282 | 0.113– 0.451 | 0.001 | 0.282 | 0.113– 0.452 | 0.001 |
| Household: couple, no children [living alone] | 0.081 | -0.011– 0.172 | 0.084 | 0.080 | -0.011– 0.172 | 0.086 |
| Household: couple and children [living alone] | 0.047 | -0.066– 0.159 | 0.417 | 0.046 | -0.066– 0.159 | 0.419 |
| Household: other [living alone] | 0.016 | -0.113– 0.145 | 0.807 | 0.015 | -0.113– 0.144 | 0.814 |
| Monthly income | 0.052 | 0.024– 0.079 | <0.001 | 0.052 | 0.024– 0.079 | <0.001 |
| Employment: part-time [full-time] | 0.075 | -0.011– 0.160 | 0.087 | 0.074 | -0.012– 0.159 | 0.092 |
| Employment: retired [full-time] | 0.003 | -0.088– 0.095 | 0.948 | 0.003 | -0.089– 0.094 | 0.952 |
| Employment: not working [full-time] | -0.345 | -0.437–-0.252 | <0.001 | -0.345 | -0.438–-0.252 | <0.001 |
| Health insurance: private [public] | 0.310 | 0.184– 0.435 | <0.001 | 0.309 | 0.184– 0.435 | <0.001 |
| Doctor visits | -0.090 | -0.094–-0.086 | <0.001 | -0.090 | -0.094–-0.086 | <0.001 |
| Hospital stays | -0.135 | -0.151–-0.118 | <0.001 | -0.135 | -0.151–-0.118 | <0.001 |
| Moved: yes [no] | -0.033 | -0.128– 0.062 | 0.498 | -0.032 | -0.127– 0.063 | 0.503 |
| 2SFCA hospitals | -0.010 | -0.049– 0.028 | 0.594 | -0.016 | -0.061– 0.029 | 0.484 |
| 2SFCA GPs | 0.018 | -0.018– 0.053 | 0.326 | 0.043 | -0.001– 0.086 | 0.056 |
| **2SFCA hospitals*Onset: yes [no]** |  |  |  | 0.010 | -0.037– 0.057 | 0.670 |
| **2SFCA GPs*Onset: yes [no]** |  |  |  | -0.046 | -0.093– 0.001 | 0.057 |
| Individual-level random intercept | Yes | | | Yes | | |
| Individual-level random slopes | Yes | | | Yes | | |
| N Observations | 36,463 | | | 36,463 | | |
| N Individuals | 3,847 | | | 3,847 | | |

*Note*: Time-to-onset = pre-onset trend effect; Onset: yes = immediate level effect at onset; Time-to-onset*Onset: yes = post-onset trend change effect. Continuous variables are mean standardised. *Abbreviations*: CI, Confidence Interval; 2SFCA, two step floating catchment area method; GP, general practitioner.

Table S2: Full Regression Results – Life Satisfaction

|  | **Onset Model** | | | **Access Model** | | |
| --- | --- | --- | --- | --- | --- | --- |
|  | **Estimate** | **95%-CI** | **p-value** | **Estimate** | **95%-CI** | **p-value** |
| Intercept | 6.478 | 6.346– 6.611 | <0.001 | 6.478 | 6.345– 6.610 | <0.001 |
| **Time-to-onset** | 0.004 | -0.007– 0.015 | 0.469 | 0.004 | -0.007– 0.015 | 0.471 |
| **Onset: yes [no]** | -0.184 | -0.239–-0.129 | <0.001 | -0.184 | -0.239–-0.129 | <0.001 |
| **Time-to-onset*Onset: yes [no]** | 0.026 | 0.011– 0.041 | <0.001 | 0.026 | 0.011– 0.041 | <0.001 |
| Age | -0.730 | -1.027–-0.433 | <0.001 | -0.731 | -1.028–-0.433 | <0.001 |
| Age² | 0.797 | 0.499– 1.095 | <0.001 | 0.798 | 0.499– 1.096 | <0.001 |
| Sex: female [male] | 0.058 | -0.030– 0.146 | 0.194 | 0.058 | -0.030– 0.146 | 0.195 |
| Years in sample | 0.034 | -0.006– 0.074 | 0.095 | 0.034 | -0.006– 0.074 | 0.095 |
| Year of onset: 2013 [2011] | 0.132 | -0.005– 0.269 | 0.059 | 0.132 | -0.005– 0.269 | 0.060 |
| Year of onset: 2015 [2011] | 0.378 | 0.254– 0.503 | <0.001 | 0.378 | 0.254– 0.503 | <0.001 |
| Year of onset: 2017 [2011] | 0.459 | 0.324– 0.595 | <0.001 | 0.459 | 0.324– 0.595 | <0.001 |
| Year of onset: 2019 [2011] | 0.504 | 0.358– 0.649 | <0.001 | 0.504 | 0.358– 0.649 | <0.001 |
| Household: couple, no children [living alone] | 0.418 | 0.341– 0.495 | <0.001 | 0.419 | 0.342– 0.495 | <0.001 |
| Household: couple and children [living alone] | 0.389 | 0.295– 0.483 | <0.001 | 0.389 | 0.295– 0.483 | <0.001 |
| Household: other [living alone] | 0.005 | -0.103– 0.112 | 0.932 | 0.005 | -0.103– 0.112 | 0.930 |
| Monthly income | 0.075 | 0.052– 0.098 | <0.001 | 0.075 | 0.052– 0.098 | <0.001 |
| Employment: part-time [full-time] | 0.054 | -0.017– 0.126 | 0.135 | 0.055 | -0.016– 0.126 | 0.131 |
| Employment: retired [full-time] | -0.003 | -0.079– 0.074 | 0.948 | -0.002 | -0.079– 0.074 | 0.949 |
| Employment: not working [full-time] | -0.443 | -0.520–-0.366 | <0.001 | -0.442 | -0.519–-0.365 | <0.001 |
| Health insurance: private [public] | 0.388 | 0.282– 0.495 | <0.001 | 0.388 | 0.282– 0.495 | <0.001 |
| Doctor visits | -0.031 | -0.035–-0.028 | <0.001 | -0.031 | -0.035–-0.028 | <0.001 |
| Hospital stays | -0.053 | -0.067–-0.039 | <0.001 | -0.053 | -0.067–-0.039 | <0.001 |
| Moved: yes [no] | 0.051 | -0.028– 0.131 | 0.204 | 0.051 | -0.028– 0.130 | 0.206 |
| 2SFCA hospitals | -0.006 | -0.039– 0.026 | 0.693 | -0.011 | -0.048– 0.026 | 0.552 |
| 2SFCA GPs | 0.004 | -0.026– 0.033 | 0.809 | 0.001 | -0.034– 0.036 | 0.961 |
| **2SFCA hospitals*Onset: yes [no]** |  |  |  | 0.010 | -0.028– 0.048 | 0.596 |
| **2SFCA GPs*Onset: yes [no]** |  |  |  | 0.006 | -0.032– 0.043 | 0.770 |
| Individual-level random intercept | Yes | | | Yes | | |
| Individual-level random slopes | Yes | | | Yes | | |
| N Observations | 36,463 | | | 36,463 | | |
| N Individuals | 3,847 | | | 3,847 | | |

*Note*: Time-to-onset = pre-onset trend effect; Onset: yes = immediate level effect at onset; Time-to-onset*Onset: yes = post-onset trend change effect. Continuous variables are mean standardised. *Abbreviations*: CI, Confidence Interval; 2SFCA, two step floating catchment area method; GP, general practitioner.

Table S3: Sensitivity Analyses – Regional Effects

|  | **Health Satisfaction** | | | **Life Satisfaction** | | |
| --- | --- | --- | --- | --- | --- | --- |
|  | **Estimate** | **95%-CI** | **p-value** | **Estimate** | **95%-CI** | **p-value** |
| Intercept | 6.110 | 5.949– 6.272 | <0.001 | 6.554 | 6.416– 6.692 | <0.001 |
| **Time-to-onset** | -0.067 | -0.082–-0.053 | <0.001 | -0.003 | -0.015– 0.009 | 0.600 |
| **Onset: yes [no]** | -0.386 | -0.454–-0.317 | <0.001 | -0.186 | -0.241–-0.131 | <0.001 |
| **Time-to-onset*Onset: yes [no]** | 0.047 | 0.028– 0.065 | <0.001 | 0.024 | 0.009– 0.040 | 0.002 |
| Age | -1.293 | -1.637–-0.949 | <0.001 | -0.706 | -1.002–-0.411 | <0.001 |
| Age² | 1.051 | 0.706– 1.396 | <0.001 | 0.774 | 0.478– 1.071 | <0.001 |
| Sex: female [male] | -0.100 | -0.201– 0.002 | 0.054 | 0.057 | -0.030– 0.144 | 0.198 |
| Years in sample | 0.145 | 0.099– 0.192 | <0.001 | 0.041 | 0.001– 0.081 | 0.043 |
| Year of onset: 2013 [2011] | -0.010 | -0.169– 0.149 | 0.905 | 0.116 | -0.020– 0.253 | 0.095 |
| Year of onset: 2015 [2011] | 0.087 | -0.058– 0.233 | 0.240 | 0.332 | 0.207– 0.458 | <0.001 |
| Year of onset: 2017 [2011] | 0.150 | -0.011– 0.310 | 0.067 | 0.402 | 0.263– 0.540 | <0.001 |
| Year of onset: 2019 [2011] | 0.220 | 0.046– 0.394 | 0.013 | 0.429 | 0.280– 0.579 | <0.001 |
| Household: couple, no children [living alone] | 0.082 | -0.009– 0.174 | 0.078 | 0.418 | 0.342– 0.495 | <0.001 |
| Household: couple and children [living alone] | 0.036 | -0.077– 0.148 | 0.532 | 0.381 | 0.287– 0.475 | <0.001 |
| Household: other [living alone] | 0.011 | -0.118– 0.140 | 0.869 | -0.003 | -0.111– 0.104 | 0.953 |
| Monthly income | 0.050 | 0.022– 0.077 | <0.001 | 0.073 | 0.050– 0.096 | <0.001 |
| Employment: part-time [full-time] | 0.070 | -0.016– 0.156 | 0.109 | 0.049 | -0.022– 0.120 | 0.176 |
| Employment: retired [full-time] | 0.001 | -0.091– 0.092 | 0.987 | -0.004 | -0.080– 0.072 | 0.920 |
| Employment: not working [full-time] | -0.347 | -0.440–-0.255 | <0.001 | -0.446 | -0.523–-0.369 | <0.001 |
| Health insurance: private [public] | 0.292 | 0.166– 0.417 | <0.001 | 0.361 | 0.254– 0.468 | <0.001 |
| Doctor visits | -0.090 | -0.094–-0.086 | <0.001 | -0.032 | -0.035–-0.028 | <0.001 |
| Hospital stays | -0.134 | -0.151–-0.118 | <0.001 | -0.053 | -0.067–-0.039 | <0.001 |
| Moved: yes [no] | -0.031 | -0.126– 0.064 | 0.522 | 0.053 | -0.026– 0.132 | 0.191 |
| 2SFCA hospitals | -0.013 | -0.052– 0.026 | 0.500 | -0.008 | -0.041– 0.025 | 0.643 |
| 2SFCA GPs | 0.019 | -0.017– 0.054 | 0.298 | 0.005 | -0.025– 0.035 | 0.728 |
| District unemployment rate | -0.030 | -0.082– 0.023 | 0.265 | -0.094 | -0.139–-0.049 | <0.001 |
| District population density | -0.049 | -0.136– 0.037 | 0.265 | 0.092 | 0.012– 0.171 | 0.025 |
| District GDP per capita | 0.102 | 0.037– 0.167 | 0.002 | -0.014 | -0.072– 0.045 | 0.649 |
| Individual-level random intercept | Yes | | | Yes | | |
| Individual-level random slopes | Yes | | | Yes | | |
| District-level random intercept | Yes | | | Yes | | |
| N Observations | 36,463 | | | 36,463 | | |
| N Individuals | 3,847 | | | 3,847 | | |

*Note*: Time-to-onset = pre-onset trend effect; Onset: yes = immediate level effect at onset; Time-to-onset*Onset: yes = post-onset trend change effect. Continuous variables are mean standardised. *Abbreviations*: CI, Confidence Interval; 2SFCA, two step floating catchment area method; GP, general practitioner; GDP, gross domestic product.

Table S4: Sensitivity Analyses – Spatial Healthcare Access Operationalization

|  | **Health Satisfaction** | | | **Life Satisfaction** | | |
| --- | --- | --- | --- | --- | --- | --- |
|  | **Estimate** | **95%-CI** | **p-value** | **Estimate** | **95%-CI** | **p-value** |
| Intercept | 6.060 | 5.903– 6.216 | <0.001 | 6.476 | 6.343– 6.608 | <0.001 |
| **Time-to-onset** | -0.059 | -0.073–-0.045 | <0.001 | 0.004 | -0.007– 0.015 | 0.461 |
| **Onset: yes [no]** | -0.383 | -0.452–-0.314 | <0.001 | -0.183 | -0.238–-0.128 | <0.001 |
| **Time-to-onset*Onset: yes [no]** | 0.047 | 0.028– 0.065 | <0.001 | 0.026 | 0.011– 0.041 | <0.001 |
| Age | -1.310 | -1.655–-0.964 | <0.001 | -0.733 | -1.030–-0.436 | <0.001 |
| Age² | 1.072 | 0.725– 1.418 | <0.001 | 0.799 | 0.501– 1.097 | <0.001 |
| Sex: female [male] | -0.096 | -0.198– 0.006 | 0.066 | 0.059 | -0.029– 0.147 | 0.187 |
| Years in sample | 0.139 | 0.093– 0.186 | <0.001 | 0.034 | -0.006– 0.074 | 0.097 |
| Year of onset: 2013 [2011] | -0.003 | -0.162– 0.157 | 0.975 | 0.133 | -0.005– 0.270 | 0.058 |
| Year of onset: 2015 [2011] | 0.119 | -0.026– 0.263 | 0.108 | 0.382 | 0.257– 0.506 | <0.001 |
| Year of onset: 2017 [2011] | 0.194 | 0.036– 0.351 | 0.016 | 0.462 | 0.326– 0.597 | <0.001 |
| Year of onset: 2019 [2011] | 0.282 | 0.113– 0.452 | 0.001 | 0.504 | 0.359– 0.650 | <0.001 |
| Household: couple, no children [living alone] | 0.079 | -0.012– 0.171 | 0.089 | 0.418 | 0.341– 0.495 | <0.001 |
| Household: couple and children [living alone] | 0.045 | -0.068– 0.157 | 0.436 | 0.389 | 0.295– 0.483 | <0.001 |
| Household: other [living alone] | 0.015 | -0.114– 0.144 | 0.820 | 0.005 | -0.103– 0.112 | 0.932 |
| Monthly income | 0.052 | 0.024– 0.079 | <0.001 | 0.075 | 0.052– 0.098 | <0.001 |
| Employment: part-time [full-time] | 0.074 | -0.011– 0.160 | 0.089 | 0.054 | -0.017– 0.125 | 0.137 |
| Employment: retired [full-time] | 0.004 | -0.088– 0.095 | 0.932 | -0.002 | -0.078– 0.075 | 0.968 |
| Employment: not working [full-time] | -0.345 | -0.437–-0.252 | <0.001 | -0.443 | -0.520–-0.366 | <0.001 |
| Health insurance: private [public] | 0.310 | 0.184– 0.435 | <0.001 | 0.386 | 0.280– 0.493 | <0.001 |
| Doctor visits | -0.090 | -0.094–-0.086 | <0.001 | -0.032 | -0.035–-0.028 | <0.001 |
| Hospital stays | -0.135 | -0.151–-0.118 | <0.001 | -0.053 | -0.067–-0.039 | <0.001 |
| Moved: yes [no] | -0.032 | -0.127– 0.063 | 0.509 | 0.051 | -0.028– 0.130 | 0.209 |
| Hospital bed density in 10km radius | -0.010 | -0.055– 0.035 | 0.659 | 0.010 | -0.028– 0.048 | 0.613 |
| GPs density in 3km radius | 0.005 | -0.036– 0.045 | 0.819 | -0.014 | -0.048– 0.020 | 0.432 |
| Distance to nearest hospital | -0.007 | -0.052– 0.039 | 0.776 | -0.019 | -0.057– 0.019 | 0.335 |
| Distance to nearest GP | 0.014 | -0.034– 0.062 | 0.575 | 0.011 | -0.029– 0.052 | 0.578 |
| Individual-level random intercept | Yes | | | Yes | | |
| Individual-level random slopes | Yes | | | Yes | | |
| N Observations | 36,463 | | | 36,463 | | |
| N Individuals | 3,847 | | | 3,847 | | |

*Note*: Time-to-onset = pre-onset trend effect; Onset: yes = immediate level effect at onset; Time-to-onset*Onset: yes = post-onset trend change effect. Continuous variables are mean standardised. *Abbreviations*: CI, Confidence Interval; 2SFCA, two step floating catchment area method; GP, general practitioner.
